# Dr. M’Cormac on Prolapsus Ani

**Published:** 1843-09

**Authors:** 


*Dr. M'Cormac on Prolapsus Ani.*—Reflecting on Dupuytren's procedure, it occurred to me that the same result might in a measure, at least while the child was at stool, be secured by careful manual traction. I immediately stated my views to the intelligent mother; she entered into them at once, and promised, if possible, to carry them into effect. According, when the child went next to stool, the skin exterior to the anus was drawn to one side by means of the fingers extended around. The little girl submitted to this with some reluctance, and complained that she could not evacuate her bowels. She was encouraged, however; a stool was obtained; from that day and date, now a month since, the bowel has not once descended. The stools, which previously were from two to four every day, have become much fewer, as well as of a more formed consistence and natural colour; while the child's health, spirits, and strength, are in other respects much ameliorated. There is now no prospect of the disease ever returning; the little girl requires comparatively little attendance; her mother, in fact, is only required to stand by, and in a short time, it is to be hoped, her onerous and anxious ministry will wholly cease.

As prolapsus ani is a very common affection coming frequently under the notice of every practitioner, and hitherto most difficult to remedy, this painless and bloodless mode of treatment, which, it is reasonable te suppose. would be equally efficient in other cases, in relieving the sufferings of helpless childhood, and lessening a mother's cares, seems deserving of attention.—*Dublin. Journ.—Medical Examiner.*

